# Serial block‐face scanning electron microscopy of erythrocytes protruding through the human placental syncytiotrophoblast

**DOI:** 10.1111/joa.12658

**Published:** 2017-07-17

**Authors:** Eleni Palaiologou, Patricia Goggin, David S. Chatelet, Emma M. Lofthouse, Christopher Torrens, Bram G. Sengers, Jane K. Cleal, Anton Page, Rohan M. Lewis

**Affiliations:** ^1^ Faculty of Medicine University of Southampton Southampton UK; ^2^ Faculty of Medicine Biomedical Imaging Unit University of Southampton Southampton UK; ^3^ Institute for life Sciences University of Southampton Southampton UK; ^4^ Faculty of Engineering and the Environment University of Southampton Southampton UK

**Keywords:** 3D imaging, epithelial barrier, placenta

## Abstract

The syncytiotrophoblast forms a continuous barrier between the maternal and fetal circulations. Here we present a serial block‐face scanning electron microscopy (SBFSEM) study, based on a single image stack, showing pooling of fetal blood underneath a region of stretched syncytiotrophoblast that has become detached from the basement membrane. Erythrocytes are protruding from discrete holes in the syncytiotrophoblast suggesting that, under specific circumstances, the syncytiotrophoblast may be permeable to fetal cells. This observation represents a pathological process but it poses questions about the physical properties and permeability of the syncytiotrophoblast and may represent an early stage in the formation of fibrin deposits in areas of syncytial denudation. This study also illustrates how the 3D images generated by SBFSEM allow the interpretation of structures that could not be understood from a single histological section.

## Introduction

The human placenta is monochorionic and haemochorial, and its syncytiotrophoblast forms a continuous barrier between the maternal and fetal circulations (Sibley, 2009). Despite the continuous nature of the syncytiotrophoblast, there is functional evidence for a size‐selective paracellular route (Sibley, 2009; Bain et al. 1990). The anatomical basis for the paracellular route remains unclear, and although trans‐trophoblastic channels have been proposed, they have not been observed to cross the full width of the syncytiotrophoblast. However, partial channels may have been observed under pressure (Kertschanska et al. 1997). Alternatively, evidence exists for regions of syncytial denudation that may allow paracellular diffusion (Brownbill et al. 2000). These regions of syncytial denudation are covered in fibrin deposits whose pathogenesis is not entirely understood. The passive permeability of the placenta decreases with increasing molecular size and proteins and cells would not be expected to cross the placenta (Bain et al. 1990; Sibley, 2009). Maternal microchimerism demonstrates that non‐trophoblastic fetal cells can enter the mother's circulation (Bianchi et al. 1996). In this study, we present evidence that fetal cells could, under specific pathological conditions, cross the syncytiotrophoblast.

## Methods

Tissue was collected after vaginal delivery from term placenta from an uncomplicated pregnancy with written informed consent and ethical approval from the Southampton and Southwest Hampshire Local Ethics Committee (11/SC/0529).

Within 30 min of delivery, a villous sample was dissected out and placed directly into 3% glutaraldehyde in 0.1 m cacodylate buffer at pH 7.4 at room temperature (RT) and stored at 4 °C until processing. Samples were treated twice with 0.1 m sodium cacodylate buffer pH 7.4 containing 0.23 m sucrose and 2 mm CaCl_2_ for 10 min, 4% OsO_4_ for 60 min, thiocarbohydrazide for 20 min, 2% OsO_4_ for 30 min and finally 2% uranyl acetate for 60 min. Samples were embedded in Spurr's resin, polymerised at 60 °C for 16 h, blocks were trimmed (100 μm^2^), mounted on an aluminium pin with conductive glue and sputter‐coated with gold/palladium (Holcomb et al. 2013). Blocks were imaged using a Gatan 3View inside an FEI Quanta 250 FEGSEM at 3.0 KV accelerating voltage and with a vacuum level of 40 Pa. The voxel size was 22 × 22 × 50 nm, and the total image size was 3000 × 3000 pixels.

Images were processed in fiji (version 2.0.0‐rc‐43) using Gaussian blur (sigma radius 2) and enhance‐ contrast (0.4% saturated pixels) (Schindelin et al. 2012). To estimate the size of the holes in the syncytiotrophoblast from which erythrocytes were protruding, the maximal diameter on the X–Y axis was measured in fiji along with three to five slices either side to ensure it was the maximum point. The maximal diameter in the Z axis was estimated by counting the number of 50‐nm slices in which it appeared. The image stack was segmented in amira (Version 6.1.1; FEI, UK) using thresholding.

The most prominent protruding erythrocyte and the surrounding region of syncytiotrophoblast were manually segmented. The surface area of the syncytiotrophoblast and the cross‐sectional area of the first slice were measured using amira. Before calculating the surface area of the syncytiotrophoblast, smoothing was performed to make it comparable to the flat surface of the base.

## Results

**Figure 1:**
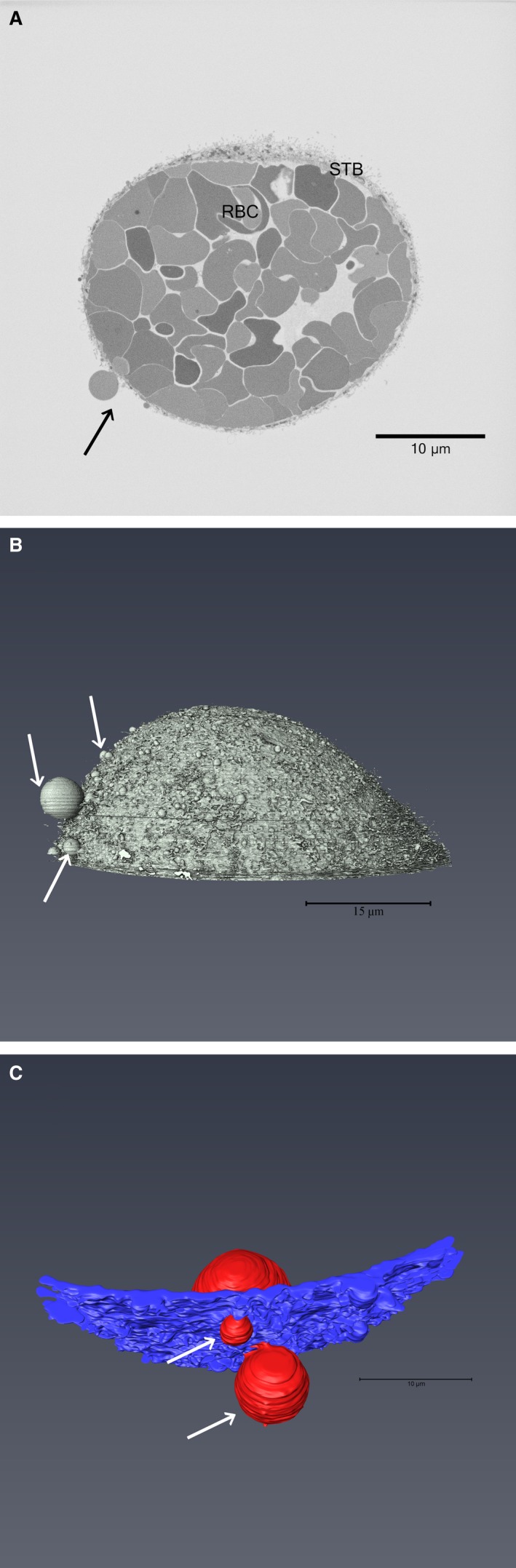
Electron microscopy image of the structure and 3D reconstructions of the stack. (A) An image from the SBFSEM showing the syncytiotrophoblast (STB), the erythrocytes (RBC) and the main protruding erythrocyte (arrow). (B) The segmented structure showing the surface of the syncytiotrophoblast and the protruding erythrocytes (arrows) (Supporting Information Video S1b). (C) Segmentation of the largest protruding erythrocyte (red) pushing through the syncytiotrophoblast (blue) in two places (arrows). (Supporting Information Video S1c).

SBFSEM revealed a region of syncytiotrophoblast detached from the underlying basement membrane. This stack consisted of 426 sequential slices with a Z resolution of 50 nm (Supporting Information Video S1a). The external face of the structure had microvilli, indicating it was the maternal‐facing microvillous membrane; the space enclosed by the syncytiotrophoblast contained 78 erythrocytes and one unidentified cell of low electron density (Fig. 1). No endothelium or stroma was apparent on the fetal side of the syncytiotrophoblast nor was there evidence of fibrin deposition. The structure was 19 μm at its deepest point, with an average diameter at the base of 42.9 μm and a circumference of 144.4 μm. Seventeen erythrocytes were observed partially protruding through the syncytiotrophoblast at 25 sites, all located on the side of the structure where the syncytiotrophoblast was thinnest (Fig, 1B & C, Supporting Information Video S1b & S1c). The median diameter of the holes was 0.56 μm (range 0.34–1.41 μm) in the X–Y plane and 0.50 μm (range 0.20–1.85 μm) in the Z plane. In 20 cases the protrusion was covered by syncytiotrophoblast membrane on the maternal side, suggesting that it may have pushed through a relatively weaker area rather than a pre‐existing hole. The surface area of the syncytiotrophoblast was 5415 μm^2^ compared with the area of the base of the structure of 1628 μm^2^, suggesting a 3.3‐fold stretch (actual stretch may be less if syncytiotrophoblast is pulled in from adjacent regions).

## Discussion

This study suggests that the syncytiotrophoblast is distensible and that, under specific pathological conditions, fetal cells can cross the syncytiotrophoblast. We speculate that this structure, which is similar to a blood blister, may represent an early stage in the formation of regions of syncytial denudation. Furthermore, the image stack highlights the value of 3D imaging, as this interesting structure would be difficult to interpret in 2D and may have been overlooked.

We interpret this structure as a pathological feature resulting from leakage from a fetal capillary (not observed) and fetal blood pooling under the syncytiotrophoblast, stretching it away from the basement membrane. Fetal hydrostatic pressure has then pushed erythrocytes through weaker areas in the stretched syncytiotrophoblast.

The structure suggests the association between the syncytiotrophoblast and basement membrane is relatively weak, consistent with observations of denuded regions of villi (Brownbill et al. 2000). It also suggests that the syncytiotrophoblast can stretch, potentially allowing movement of villi. The only holes observed in the syncytiotrophoblast were those with protruding erythrocytes, suggesting that they only open under higher pressures. The holes were smaller than the protruding regions of erythrocyte, indicating that they had sufficient structural integrity to prevent a rip occurring. These holes could represent regions of damage, trans‐syncytial channels or a structural feature making the syncytiotrophoblast locally weak.

A limitation of this study is that it is a single observation and that the underlying villus was not observed. This is an inherent limitation of relatively low throughput techniques such as SBFSEM, but the image may help the interpretation of such structures when they are observed in 2D. Another issue is whether shrinkage could have caused the erythrocytes to push through the membrane. However, we do not think this is likely, as shrinkage would primarily occur during dehydration, which occurs after the tissues are hardened due to aldehyde fixation.

Across the villi there are regions of damage in which fibrin deposits replace the trophoblast (Nelson et al. 1990). These could form where the trophoblast is sheared off directly or in regions where blood has pooled below the trophoblast and clotted before the overlying trophoblast died. Although there is no evidence of fibrin deposits in this image, it is possible that this represents an early stage in the evolution of fibrin deposits. Understanding the pathogenesis of these regions is important as they may mediate paracellular diffusion of small solutes (Brownbill et al. 2000).

This observation probably represents a pathological feature, but it does demonstrate a potential route for transfer of fetal cells to the mother through the syncytiotrophoblast. This could explain maternal microchimerism, where fetal lymphoid cells persist in the mother's body (Bianchi et al. 1996). This image raises questions about the strength and permeability of the syncytiotrophoblast and may represent an early stage in the formation of fibrin deposits in areas of syncytial denudation.

## Author contributions

RL, BS, JC and CT designed the study and were involved in the provision of financial and maternal resources. EP, EL, PG, CP and AP conducted sample collection, processing and data acquisition. EP and DC performed image analysis. EP, RL, DC and PG composed the initial manuscript. All the authors revised the manuscript and approved the final draft.

## Supporting information


**Video S1a.** A movie showing all of the slices in the stack in sequence.

**Video S1b.** A movie showing a 3D reconstruction of the structure focused on the microvillus surface of the syncytiotrophoblast and the protruding erythrocytes, which can be seen as spheres on the surface.

**Video S1c.** A movie showing the largest protruding erythrocyte in red and the region of syncytiotrophoblast around it in blue.
